# Proteomic responses to elevated ocean temperature in ovaries of the ascidian *Ciona intestinalis*

**DOI:** 10.1242/bio.024786

**Published:** 2017-05-12

**Authors:** Chelsea E. Lopez, Hannah C. Sheehan, David A. Vierra, Paul A. Azzinaro, Thomas H. Meedel, Niall G. Howlett, Steven Q. Irvine

**Affiliations:** 1 Departments of Biological Sciences, University of Rhode Island, Kingston, RI 02881, USA; 2 Cell and Molecular Biology, University of Rhode Island, Kingston, RI 02881, USA; 3 Biology Department, Rhode Island College, Providence, RI 02908, USA

**Keywords:** Climate change, Gametogenesis, Ecological physiology, Invertebrate reproduction

## Abstract

*Ciona intestinalis*, a common sea squirt, exhibits lower reproductive success at the upper extreme of the water temperatures it experiences in coastal New England. In order to understand the changes in protein expression associated with elevated temperatures, and possible response to global temperature change, we reared *C. intestinalis* from embryos to adults at 18°C (a temperature at which they reproduce normally at our collection site in Rhode Island) and 22°C (the upper end of the local temperature range). We then dissected ovaries from animals at each temperature, extracted protein, and measured proteomic levels using shotgun mass spectrometry (LC-MS/MS). 1532 proteins were detected at a 1% false discovery rate present in both temperature groups by our LC-MS/MS method. 62 of those proteins are considered up- or down-regulated according to our statistical criteria. Principal component analysis shows a clear distinction in protein expression pattern between the control (18°C) group and high temperature (22°C) group. Similar to previous studies, cytoskeletal and chaperone proteins are upregulated in the high temperature group. Unexpectedly, we find evidence that proteolysis is downregulated at the higher temperature. We propose a working model for the high temperature response in *C. intestinalis* ovaries whereby increased temperature induces upregulation of signal transduction pathways involving PTPN11 and CrkL, and activating coordinated changes in the proteome especially in large lipid transport proteins, cellular stress responses, cytoskeleton, and downregulation of energy metabolism.

## Abstract (precis)

**Summary:** Shotgun proteomics study of the effect of chronic elevated water temperature identifying 62 differentially expressed ovarian proteins and a model for the proteomic response mediated by PTPN11 and CrkL.

## INTRODUCTION

Marine environments are changing rapidly due to the increase in greenhouse gases in the atmosphere. Seawater temperatures are projected to rise by as much as 4°C by the end of the century, and other effects such as ocean acidification and larger regions of hypoxia are also anticipated (Meinshausen et al., 2011; Allison and Bassett, 2015; Hare et al., 2016).

A major component of fitness, that should determine in large part whether a population persists, is the ability of the reproductive system to function in a particular environment (e.g. Lawrence and Soame, 2004; Pankhurst and Munday, 2011). If the reproductive system of an organism must operate outside of its normal temperature range due to environmental change, there are three possible responses: (i) the reproductive physiology could be plastic enough for the organism to acclimate to the new condition; (ii) the organism could evolve physiological adaptations to the change; or (iii) the organism could become locally extinct. In reality, the outcome could also be some combination of these responses.

### Temperature and reproduction in *C. intestinalis*

The most extensive examination of the effect of temperature on reproduction in *C. intestinalis* is that of Dybern (1965). This study was conducted in coastal Sweden, but also discusses data from other locations. The water temperature ranges of the Swedish locations are quite similar to those of our study site in Rhode Island, USA – with a low temperature of around 0°C in late winter, and a high temperature of 20-24°C in late summer (Fig. S1). Normal development is possible above 8°C, but the percentage of normally developing embryos decreases above 20°C. We have observed a similar pattern in our study region of coastal Rhode Island, i.e. *C. intestinalis* become gravid when water temperature rises above approximately 10°C in the spring. However, the reliability of normal development from animals collected in mid to late summer decreases markedly, as water temperatures exceed 18-20°C.

In the Mediterranean, where water temperatures vary from approximately 12°C to 27°C, animals can spawn throughout the winter (Caputi et al., 2015). During the summer, however, developmental success decreases markedly at the high water temperatures (C. Sardet, personal communication). On the other hand, in regions where the water temperatures remain between the limits of 8°C and 18°C, such as South Africa and California, animals are gravid year round, and exhibit normal development within that range (Dybern, 1965, and personal observations). Thus, in *C. intestinalis* the seasonality of reproduction appears to be governed largely by temperature patterns.

These observations indicate that *C. intestinalis* has normal gametogenesis and development between approximately 8°C and 18°C. In areas where water temperatures exceed this range local adaptation has been insufficient to allow normal reproductive success during the warmest part of the year. It is not known whether some physiological constraint prevents adaptation to warmer water, or if there is just insufficient selective pressure to adapt to a wider temperature range.

While several studies have examined the effects of temperature on gene expression and physiological performance in marine invertebrates (e.g. Hofmann and Todgham, 2010; Somero, 2010, 2012; Tomanek, 2014; Bolton et al., 2013), few have been focused on the reproductive system. Because of the clear effects of elevated temperature on reproduction in *C. intestinalis*, we are interested in how protein expression changes as the environmental conditions move outside the range for normal reproduction and development. In addition, we were interested in whether use of new technology for identification and quantification of proteins, namely liquid chromatography-tandem mass spectrometry (LC-MS/MS) using an Orbitrap instrument (also known as ‘shotgun proteomics’), would give a deeper global picture of the physiological response to elevated temperature than previous gel-based methods.

Using this approach we identified more than 60 proteins up- or down-regulated between the ovaries of animals acclimated to a control versus elevated temperature. We found groups of proteins previously implicated in the response to high temperature, as well as highly expressed proteins not previously identified. Due to a wealth of genomic and reverse genetic tools, *C. intestinalis* is an ideal organism for the functional study of the physiological roles of these novel temperature-response proteins.

## RESULTS

### Experimental setup and protein expression profiling

*C. intestinalis* embryos spawned *in vitro* from gametes of a single pair of adults were reared at a control temperature of 18°C, which is known to produce viable gametes, and elevated temperature of 22°C, which is at the upper temperature range locally. Ovaries were dissected from five animals at each temperature (10 samples total). Each ovary was treated as a separate replicate for LC-MS/MS. A total of 1532 proteins were identified in samples from both temperatures at a 1% false discovery rate (FDR). Of these, 62 proteins met our criteria for differential expression (Fig. S2; Tables 1 and 2). A complete list of all 1532 protein identifications, peak area intensity measurements, and *P*-values are available in Table S1.

**Table 1.**
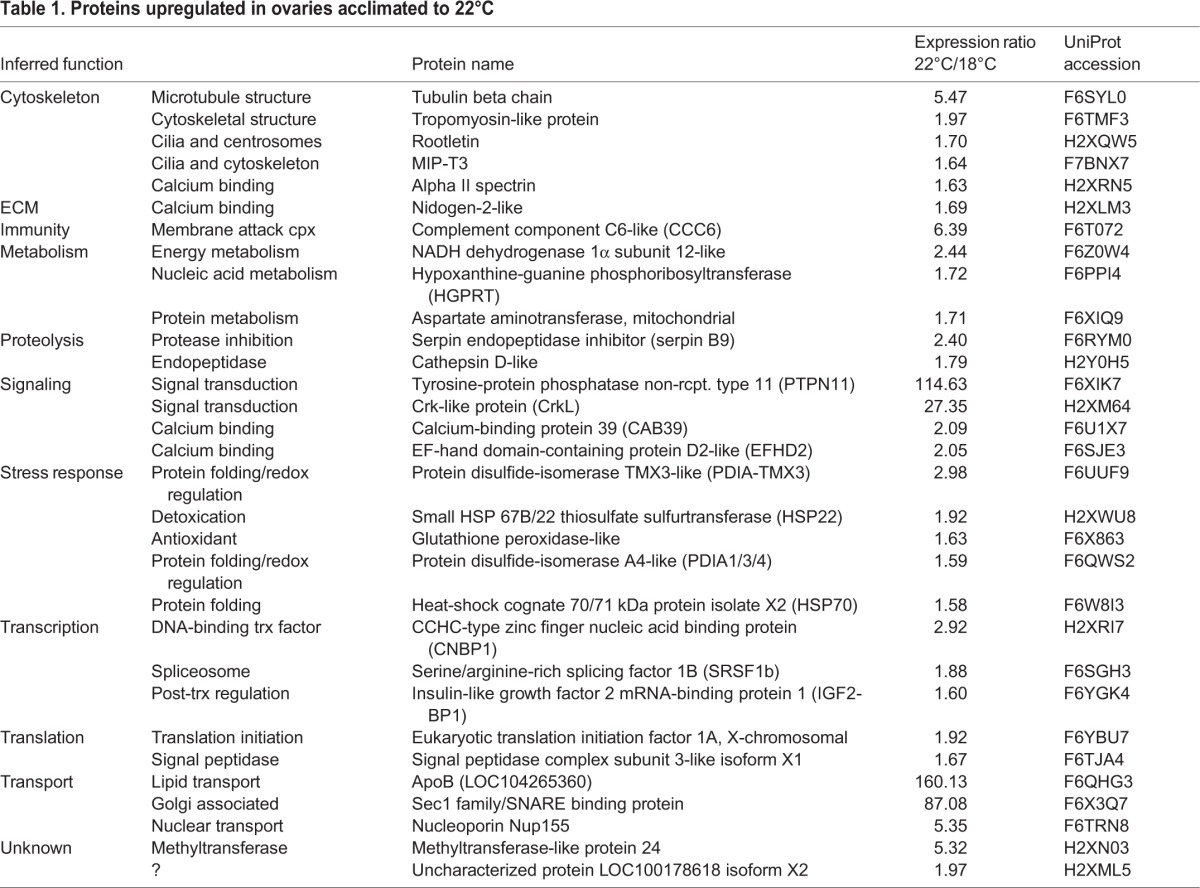
**Proteins upregulated in ovaries acclimated to 22°C**

**Table 2.**
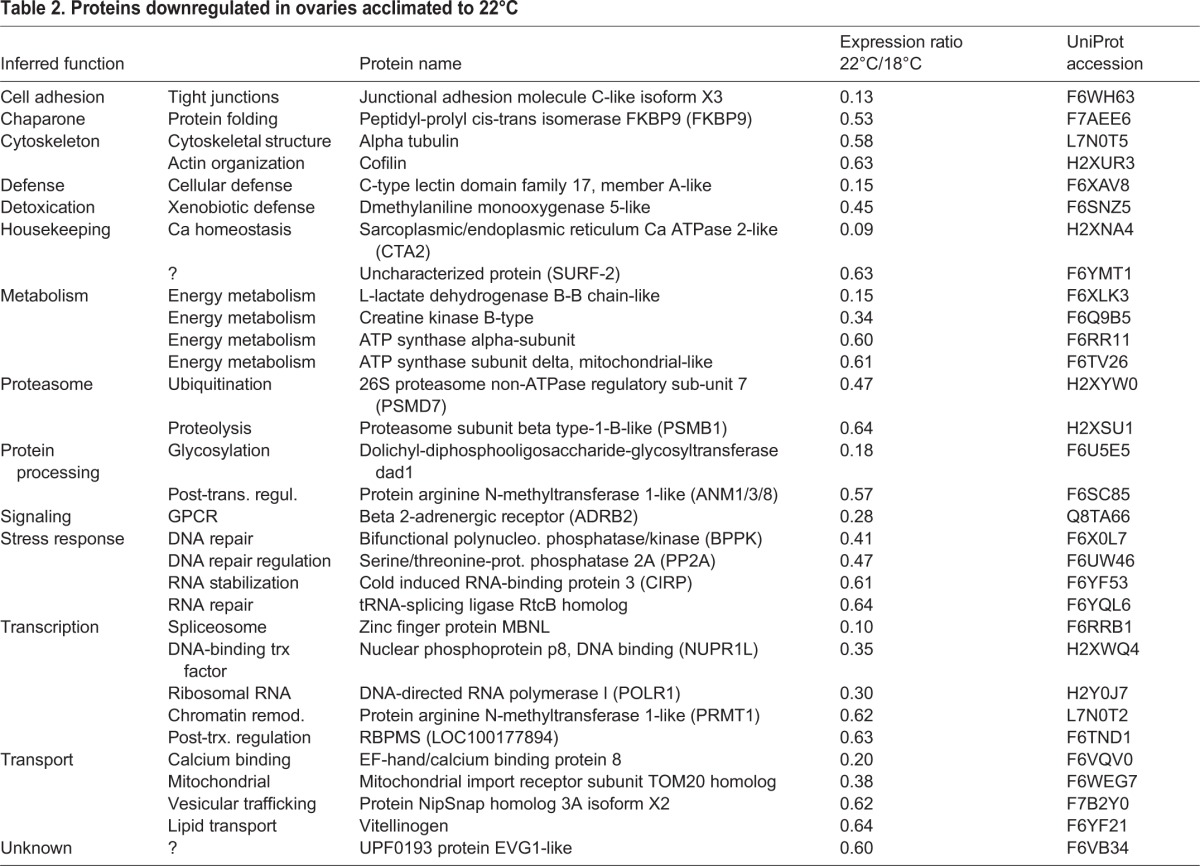
**Proteins downregulated in ovaries acclimated to 22°C**

**Fig. 1.**
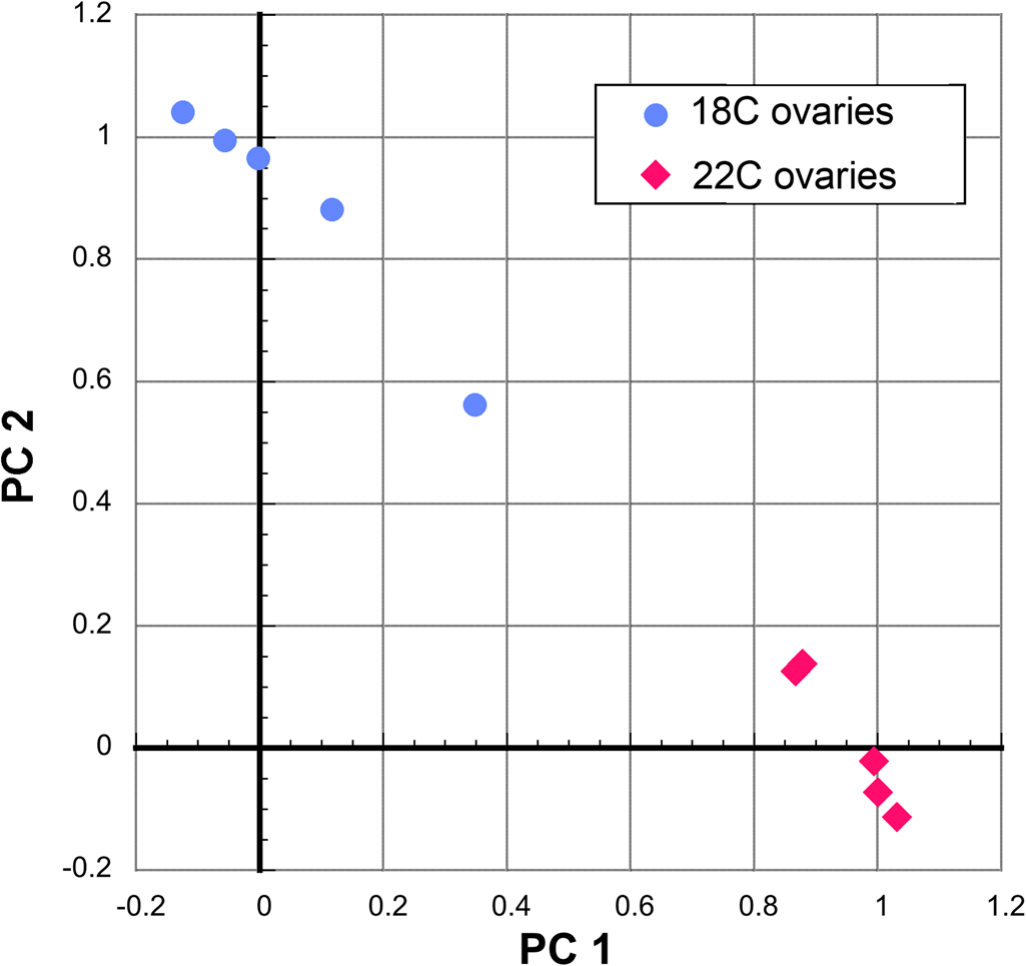
**Principal components analysis of the peak area intensity area values for 62 differentially regulated proteins.** Proteins listed in Tables 1 and 2. Principal component (PC) 1 explains 71% of the variation, while PC 2 explains an additional 19%.

Fig. 1 shows the result of a principal components analysis (PCA) performed on the expression levels of the 62 differentially regulated proteins. A similar pattern was seen in PCA on the whole 1532 protein dataset. The PCA indicates that there is wide variation for the 18°C samples. However, the 22°C samples are clustered and distinct from the 18°C data points, indicating a coordinated proteomic response to the higher temperature.

**Fig. 2.**
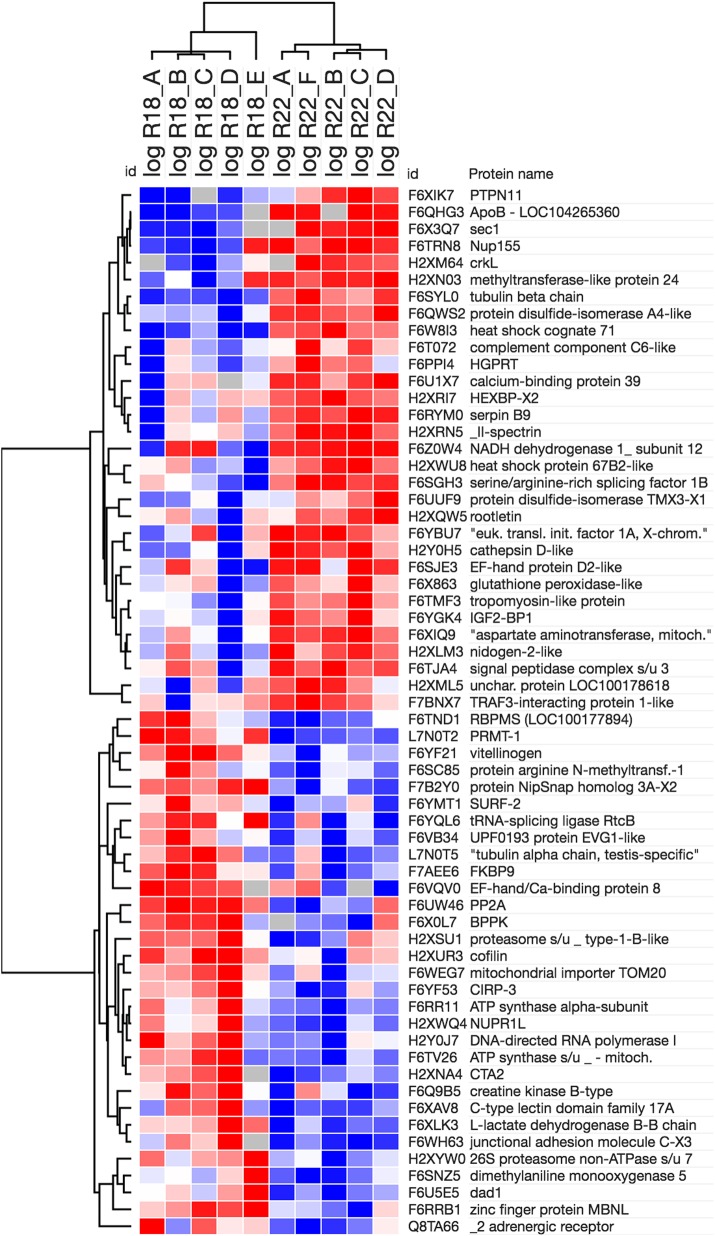
**Heat map showing Pearson clustering of 62 proteins.** Proteins listed in Tables 1 and 2. R18_A-E are ovaries from animals reared at 18°C, R22_A-E are from animals reared at 22°C.

A heat map of the 62 differentially regulated proteins shows the high variability within each condition (Fig. 2). We propose that this is due to stochasticity in the protein levels of the individuals sampled (Clark et al., 2017; Fields et al., 2012; Serafini et al., 2011), which may be at different points in overall metabolic state at the time their ovaries were processed (W. Dowd, personal communication). However, a clear distinction is apparent between the 31 proteins upregulated at 22°C, and the 31 deemed relatively downregulated in both the heat map and PCA.

### Protein over-representation analysis

**Fig. 3.**
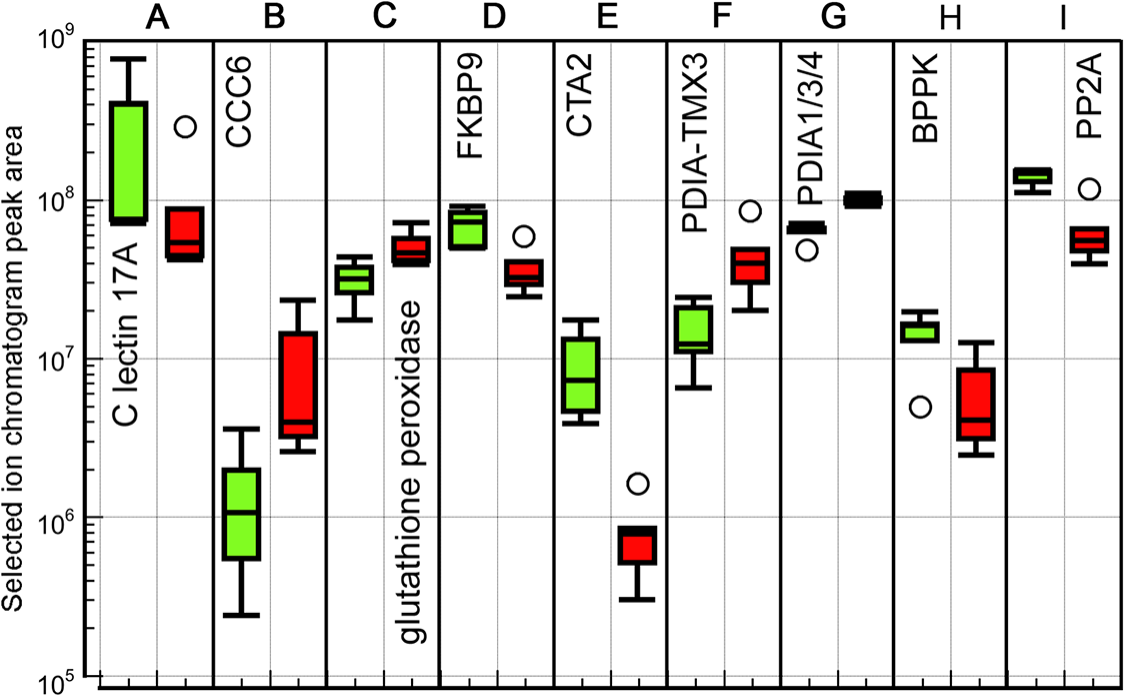
**Box plots of peak area intensity measurements for differentially expressed proteins identified in the PANTHER overrepresentation analysis.** GO categories: (A) cellular defense response; (A-C) immune system process; (D,E) cellular calcium ion homeostasis; (F-I) response to stress. 18°C samples: green (left) boxes; 22°C samples: red (right) boxes. Five biological replicates at each temperature. Boxes indicate median and interquartile (IQD) range, whiskers show top and bottom values, and circles indicate outliers more than 1.5 times the IQD from the upper or lower quartiles.

PANTHER was used to estimate over- and under-represented gene ontology (GO) classifications in the differentially expressed proteins (Table 3). All of the over-represented GO biological processes could be seen as associated with cell protective functions, with some proteins upregulated and others downregulated. The ‘cellular defense response’ and ‘immune system process’ categories are comprised of a C-type lectin, which is downregulated, and complement component C6, part of the microbial defense attack complex, and the antioxidant glutathione peroxidase, which are both upregulated (Fig. 3A-C). The other over-represented categories, ‘cellular calcium ion homeostasis’ and ‘response to stress’, similarly have some upregulated and some downregulated members. FKBP9 and CTA2 (Fig. 3D,E), which are downregulated at high temperature, are involved in both Ca ion transport and protein folding in the endoplasmic reticulum (ER). Two other protein folding enzymes, are disulfide-isomerases, which are upregulated at high temperature (PDIA-TMX3 and PDIA1/2/3; Fig. 3F,G). The other two proteins in this category, BPPK and PP2A (Fig. 3H,I) are downregulated and have functions linked to DNA repair. BPPK, bifunctional polynucleotide phosphatase/kinase, is associated with binding to damaged DNA, DNA replication, base-excision repair, and response to oxidative stress. Mutations in the *BPPK* gene have been linked to defective DNA repair in human patients (Shen et al., 2010), and the protein has been shown to rescue sensitivity to oxidative damage agents in an *E. coli* DNA repair mutant (Jilani et al., 1999)*.* PP2A, serine-threonine protein phosphatase (Fig. 3I), is also linked to DNA repair and binds to DNA double-strand breaks (DSBs) (Kalev et al., 2012). It is both a negative and positive regulator of the DSB pathway, so its downregulation at high temperature may allow activation of the DSB pathway to deal with oxidative DNA damage. On the other hand, both of these proteins may be downregulated because the cells are undergoing apoptosis or necrosis rather than repairing DNA. Taken together, these over-represented functional categories with both up- and down-regulated members suggest turnover in cell protective proteins between the lower temperature and high temperature conditions.

**Table 3.**
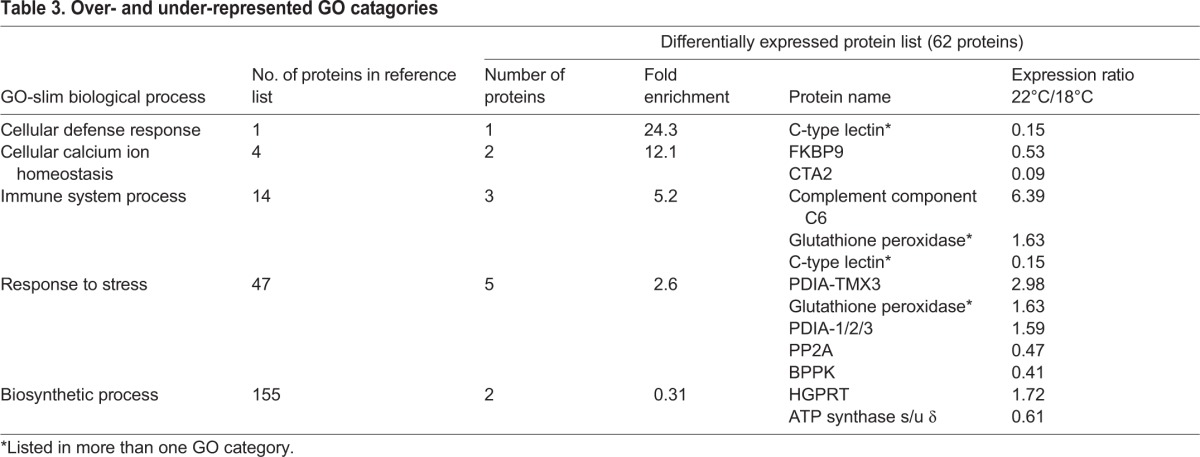
**Over- and under-represented GO catagories**

The only under-represented GO category was ‘biosynthetic process’, with just two members (Table 3). The underrepresentation of metabolic proteins in the list of those differentially regulated is consistent with the notion that the major changes in protein abundance are connected with cell protective functions.

### Other stress-response proteins and molecular chaperones

**Fig. 4.**
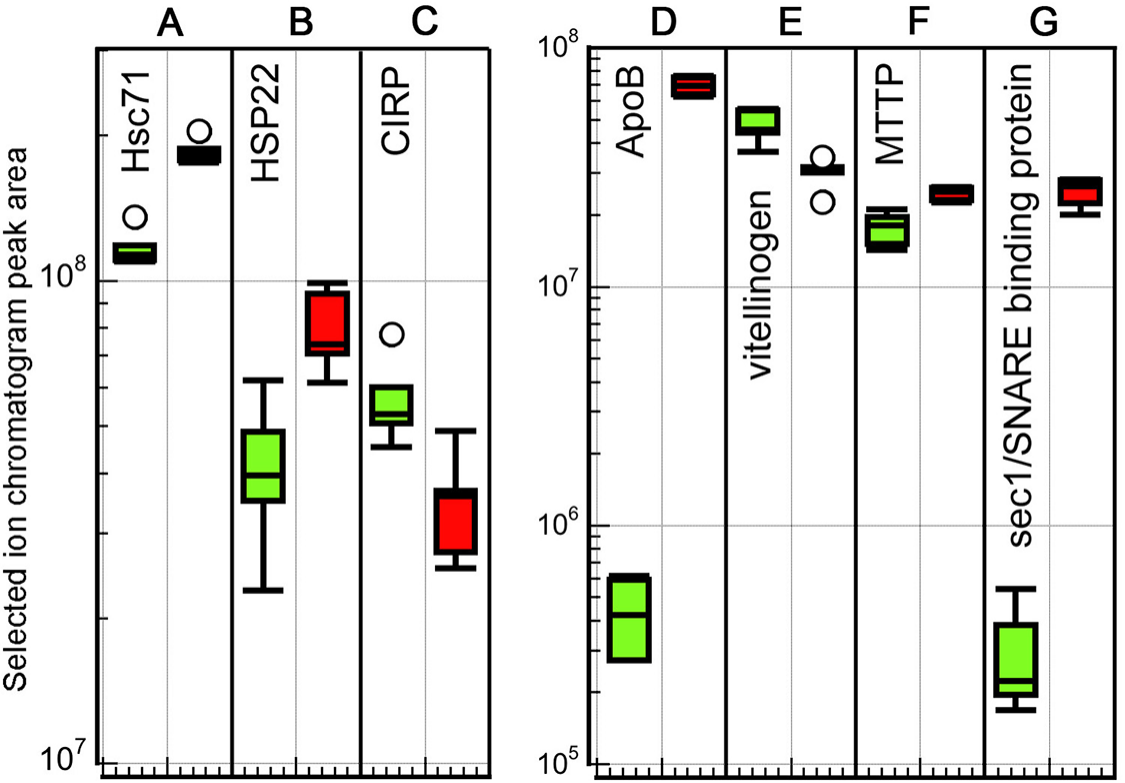
**Box plots of peak area intensity measurements for differentially expressed proteins associated with response to stress and lipid transport**. (A-C) Proteins associated with response to stress (refer also to Fig. 3F-I). (D-G) Lipid transport proteins. 18°C samples: green (left) boxes; 22°C samples: red (right) boxes. Five biological replicates at each temperature. Box plot definition as in Fig. 3 legend.

In addition to the stress-related proteins noted above, additional molecular chaperones were found to be differentially expressed, indicative of protein misfolding, and possibly a cellular stress response (CSR) (Kültz, 2005). In particular, the expression level of the putative heat shock cognate (HSC) 71, is significantly differentially expressed, with a 1.6-fold change, even when evaluated by a stringent false discovery test (Q=0.036, Fig. 4A). Since the Hsp70 family members have such high sequence similarity to each other, we cannot unambiguously assign the orthology of this protein. Regardless, the function of all the Hsp70 proteins is in aiding proper folding of proteins and elimination of those terminally misfolded (Korsloot et al., 2004; Kültz, 2005).

A putative small heat shock protein (HSP), which is similar in sequence to *Drosophila* Hsp67Bb and Hsp22, is also upregulated at 22°C (Fig. 4B). It has a rhodanese-like domain, which has sulfurtransferase function implicated in cyanide detoxication and hydrogen sulfide metabolism, and so may be more involved in cell protection than as a chaperone, although it is upregulated due to heat stress in *Drosophila* (Colinet et al., 2010).

Also downregulated in the 22°C samples is cold-induced RNA binding protein, or CIRP (Fig. 4C). CIRP inhibits DNA damage-induced apoptosis in humans (Lee et al., 2015), and has been shown to be downregulated at high temperatures (Nishiyama et al., 1998) consistent with the 1.6-fold downregulation of the *C. intestinalis* protein at 22°C. This pattern is similar to the lowered expression levels of the DNA repair associated proteins BPPK and PP2A noted above. The downregulation of CIRP may allow apoptotic pathways to operate at the higher temperature to eliminate heat damaged cells.

### Ovary-associated proteins and lipid transport

A number of proteins have been previously identified as being characteristic of ovarian proteomes (Knoll-Gellida et al., 2006; Song et al., 2006; Groh et al., 2011). Notably, by far the largest difference in protein expression between the two temperature groups was for the large lipid transport protein (LLTP) ApoB (Nomura et al., 2009; Smolenaars et al., 2007), which was 160 times upregulated in the high temperature condition (Fig. 4D). Another LLTP, vitellinogen (Fig. 4E), was conversely slightly downregulated at high temperature. The only other LLTP found in the *C. intestinalis* genome is microsomal triglyceride transfer protein (MTTP, Fig. 4F), which was upregulated at 22°C (*P*<0.01) but only by 1.3-fold (Table S1). Both vitellinogen and MTTP are expressed at roughly the upregulated level of ApoB. These data suggest that the main lipid transporters in the ovary are vitellinogen and MTTP, with ApoB only being activated at high temperature.

Two other proteins characteristic of ovaries (Knoll-Gellida et al., 2006; Groh et al., 2011) are significantly downregulated in the 22°C ovaries. One is L-lactate dehydrogenase B chain. The 6.7-fold downregulation of this protein may reflect a general reduction in metabolic protein levels at the high temperature condition, consistent with the downregulation of other energy metabolism proteins (see below). The other is cold-inducible RNA-binding protein, noted above (Fig. 4C). There are other proteins typically associated with ovaries present in the MS data that are not differentially expressed, but found as expected. These include the stem cell associated proteins piwi and vasa, along with others summarized in Table S2.

In addition to these characteristically ovary-associated proteins, another lipid transporter is found to be 87-fold upregulated at the higher temperature. This is a sec1 family/SNARE binding protein (Fig. 4G). Proteins from this family are involved in vesicle-mediated transport and secretion (Halachmi and Lev, 1996) suggesting a large increase in Golgi-associated vesicle transport, or exocytosis, or both.

### Upregulated signaling molecules

**Fig. 5.**
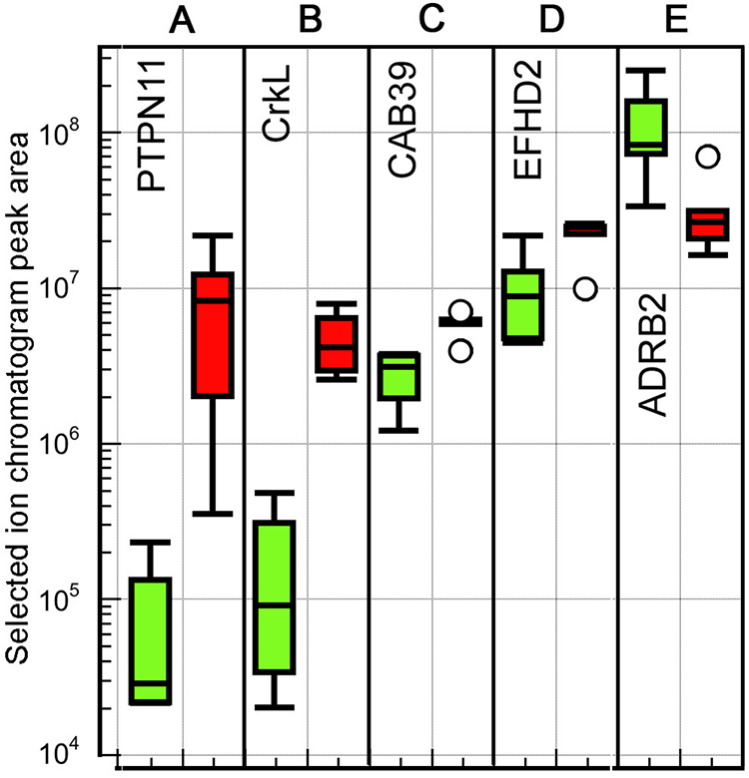
**Box plots of peak area intensity measurements for differentially expressed proteins associated with intracellular signaling.** 18°C samples: green (left) boxes; 22°C samples: red (right) boxes. Five biological replicates at each temperature. Box plot definition as in Fig. 3 legend.

Two of the most extremely upregulated proteins in the high temperature ovaries are the signal transduction factors protein tyrosine phosphatase non-receptor type 11 (PTPN11/SHP2), at 114-fold upregulated, and crk-like protein (CrkL), which is 27-fold upregulated (Fig. 5A,B). PTPN11 is both an adaptor protein and phosphatase, involved in a host of functions, most of which involve development (Neel et al., 2003). There appears to be a distinct threshold for expression, with a very low level at 18°C, and much higher at 22°C. One possible role is in lipid metabolism, which could relate to the similar radical upregulation of ApoB (Tajan et al., 2015, *cf.* Fig. 4D).

The other highly expressed signaling protein at 22°C is CrkL. This is also an adaptor protein, although non-enzymatic, that binds signal transduction proteins involved in various functions, such as phagocytosis, immune response, cell migration and cell adhesion (Feller, 2001). One known binding partner is paxillin, which is found in all of our ovary samples (F7ALM5, Table S1). Paxillin is another adaptor which organizes signal transduction complexes in focal adhesions at the plasma membrane. CrkL also binds integrins to facilitate signal transduction (Li et al., 2003). Three integrins are found in our ovary data (F6QQK4, F6RRW9, F6QBL6, Table S1). In particular beta 1-integrin has been shown by multiple studies to interact with CrkL (Mintz et al., 2009; Sattler et al., 1997; Ungewiss et al., 2016). Upregulation of CrkL may function to form a number of these signal transduction complexes as a response to the high temperature.

Calcium binding protein 39 (CAB39, Fig. 5C) is twofold upregulated in the 22°C ovaries. This is another adaptor protein that interacts with various protein kinases. Notably, one of the identified binding partners is the serine/threonine protein kinase OSR1 (Ponce-Coria et al., 2012; Gagnon and Delpire, 2012), which is present in the MS data, but not differentially expressed (F6V299, Table S1). CAB39 is capable of highly upregulating OSR1 function (Filippi et al., 2011), suggesting that it is interacting with signal transduction factors to amplify the high temperature response.

EF-hand domain-containing protein D2-like (EFHD2, Fig. 5D) is also twofold upregulated at 22°C. This is another calcium-binding adaptor protein with multiple roles (Duetting et al., 2011). The high expression of ezrin in all samples (Table S1), which is a direct target of EFHD2, is consistent with a role for this protein in regulation of cytoskeletal proteins (Chęcińska et al., 2009).

A protein most similar to the human beta 2-adrenergic receptor (ADRB2, Fig. 5H) is the most downregulated signaling factor in 22°C ovaries, at 3.6-fold. This adrenergic receptor in vertebrates is a main transducer of sympathetic nervous system responses (Johnson, 2006). It is worth noting that the absolute amount of this protein is higher than that of the other identified signaling molecules even at the downregulated level.

### Transcription and mRNA processing

**Fig. 6.**
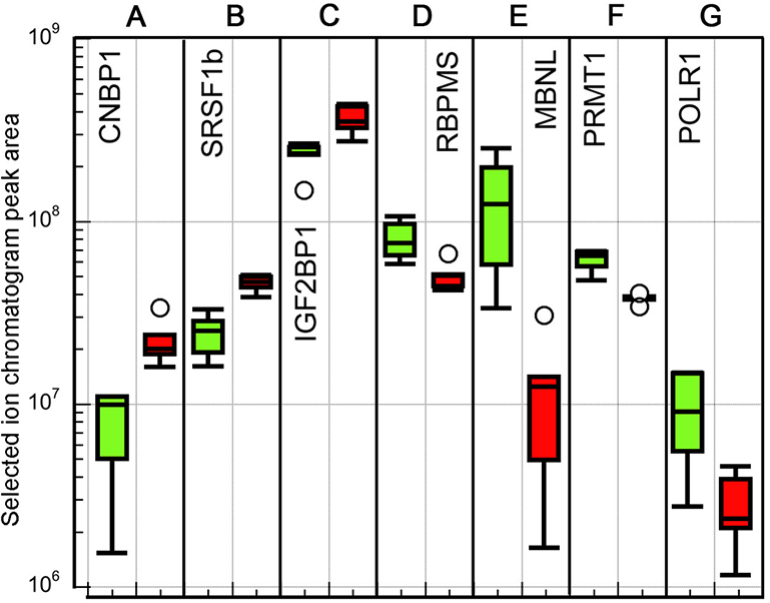
**Box plots of peak area intensity measurements for differentially expressed proteins associated with transcription.** 18°C samples: green (left) boxes; 22°C samples: red (right) boxes. Five biological replicates at each temperature. Box plot definition as in Fig. 3 legend.

Several proteins involved in transcription and post-transcriptional processes are differentially regulated at high temperature (Fig. 6). CNBP1 and SRSF1b have been implicated in regulation of both cell proliferation and apoptosis (Fig. 6A,B). Reduced levels of CNBP1, a zinc finger DNA and RNA binding protein, have been shown to reduce cell proliferation rates, either by reducing the global rate of protein synthesis, or by affecting the transcription of genes required for cell proliferation (Calcaterra et al., 2010; Antonucci et al., 2014) as well as increasing apoptosis (Weiner et al., 2007). Also interesting is the association of CNBP with stress granules in human cells (Rojas et al., 2012). SRSF1b, required for alternative splicing, is a potent proto-oncogene, which fosters cell proliferation and suppresses apoptosis when overexpressed in mammalian cells (Anczuków et al., 2012; Das and Krainer, 2014; Karni et al., 2007). Upregulation of these proteins suggest that higher temperature may induce mechanisms for increased cell proliferation in the ovary and decreased apoptosis.

Insulin-like growth factor 2 mRNA-binding protein 1 (IGF2BP1, Fig. 6C), 1.6-fold upregulated, is another multifunctional protein that regulates many mRNAs post-transcriptionally. Like CNBP, IGF2BP1 targets mRNAs to stress granules under heat stress (Stöhr et al., 2006). IGF2BP1 also has been shown to have an ovary-specific function in blocking the translation of cyclin B1 mRNA to maintain zebrafish oocytes in an immature state (Takahashi et al., 2014).

Among downregulated proteins, RBPMS (Fig. 6D) is an RNA binding protein that has nuclear, cytoplasmic, and RNA transport functions (Farazi et al., 2014). Like IGF2BP1, RBPMS has an ovary/oocyte-specific role in mRNA transport and localization to the vegetal region in *Xenopus* oocytes (Zearfoss et al., 2004). It is degraded at oocyte maturation, while overexpression accelerates oocyte maturation (Song et al., 2007). RBPMS has also been shown to interact with the germline associated protein Nanos to sequester it to germinal granules destined for the germ cell lineage (Aguero et al., 2016).

MBNL1/2/3 (Muscle-blind, Fig. 6E), tenfold downregulated at 22°C, is a zinc-finger RNA-binding protein. It has been shown in mouse and *Drosophila* to regulate both alternate splicing in the nucleus, and intracellular cytoplasmic localization through 3′-UTRs (Wang et al., 2012; Konieczny et al., 2014).

Of the two other proteins assigned to this category, PRMT1 (Fig. 6F) is a methyltransferase. Its targets include histones, and it can be either transcriptionally activating or repressing. However, it is the major asymmetric arginine-methylase and has many other targets, so its function may be in post-translational rather than transcriptional regulation (Nicholson et al., 2009). In mouse, knockdown of PRMT1 causes defective DNA double-strand break repair (Boisvert et al., 2005). This function could be needed at higher temperatures (Tomanek, 2015), so its downregulation is unexpected.

Finally, POLR1 (Fig. 6G) is the large subunit of RNA polymerase I. This protein is threefold downregulated at the 22°C condition, which should result in a proportionately lower rate of ribosome synthesis, and a concomitant reduction in protein synthesis. These data seem to be at odds with the upregulation of CNBP, SRSF1b, and IGF2BP1, all of which promote cell proliferation.

### Cytoskeleton

**Fig. 7.**
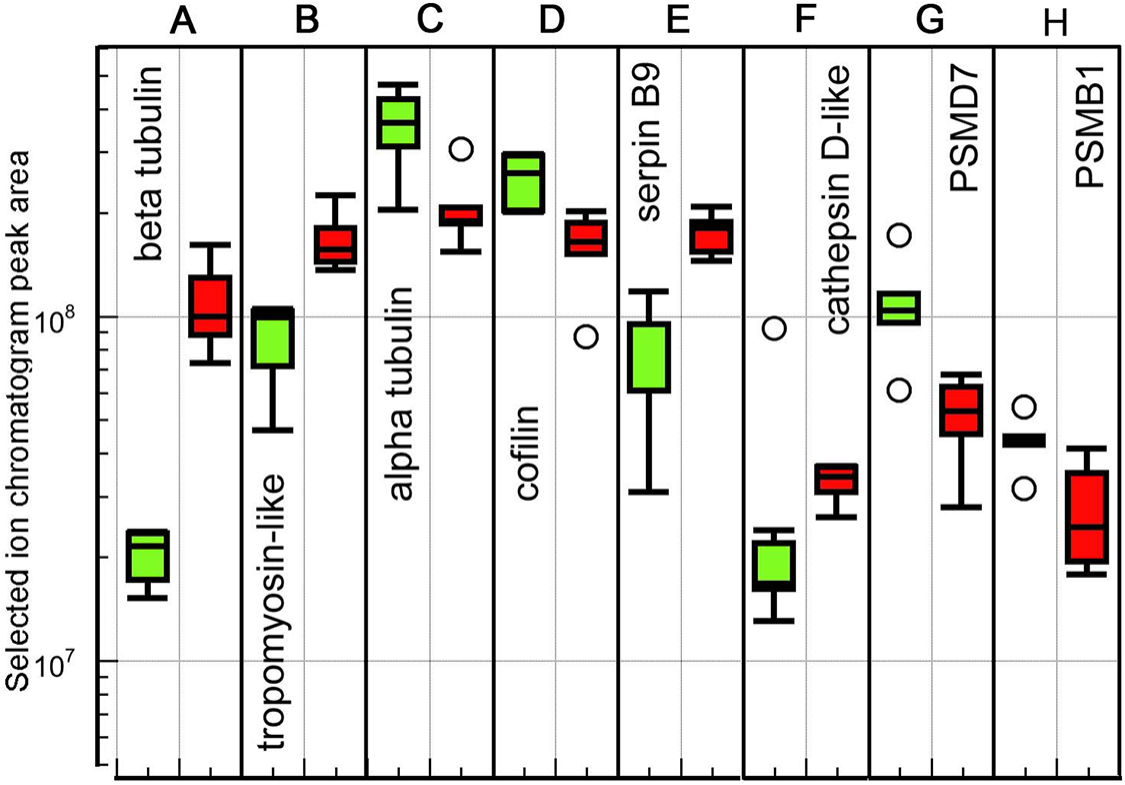
**Box plots of peak area intensity measurements for proteins associated with the cytoskeleton, proteolysis, and the proteosome.** (A-D) Cytoskeletal proteins. (E,F) Proteins associated with proteolysis, while G and H are associated with the proteasome. 18°C samples: green (left) boxes; 22°C samples: red (right) boxes. Five biological replicates at each temperature. Box plot definition as in Fig. 3 legend.

Cytoskeletal protein expression changes have been implicated in the response to elevated temperature by several other studies (Logan and Buckley, 2015; Serafini et al., 2011; Artigaud et al., 2014; Fields et al., 2012; Jayasundara et al., 2015). Our MS data identifies six isoforms of α tubulin and two of β tubulin (Table S1). Of these, one β tubulin is upregulated at 22°C (>fivefold, Fig. 7A), and one α tubulin is downregulated (1.7-fold, Fig. 7C). The other isoforms do not exhibit differential expression. If the measured quantities of the isoforms for each protein are added, the totals also are not different between conditions. Curiously, the total quantity of α tubulin isoforms is almost double that of the β tubulin isoforms, suggesting that the β tubulins are under sampled, or the α tubulin peptides are mis-mapped to proteins. However, the measurement of significant differences between particular isoforms of each chain type suggests that the composition of the microtubules, i.e. the ‘tubulin code’, differs between conditions (Janke, 2014).

Tropomyosin levels are about doubled in the 22°C ovaries (Fig. 7B). Tropomyosin associates with actin and regulates binding of myosin and intracellular transfer processes (Manstein and Mulvihill, 2016). The six actin isoforms identified in our MS data, on the other hand, do not show differential expression. It could be the case though that there are significant changes in the actin cytoskeleton due to the change in tropomyosin levels, which might also relate to the downregulation of cofilin (Fig. 7D), which depolymerizes actin (Bernstein and Bamburg, 2010). It is also possible that the upregulation of tropomyosin relates to an increase in intracellular transport through regulation of myosin binding, as reflected in the high upregulation of sec1/SNARE binding protein mentioned above.

Rootletin and MIP-T3 are upregulated by about 1.6-fold in 22°C ovaries (Table 1), and both have primary functions in cilia; rootletin in the structure of the rootlet, and MIP-T3 in intraflagellar transport (Inglis et al., 2006; Li et al., 2008; Mohan et al., 2013). These data suggest that ciliary formation and/or activity may be increased due to the higher temperature. However, rootletin also functions in mitosis (Bahe et al., 2005) and MIP-T3 has a role in TRAF signaling (Ling and Goeddel, 2000), which could be alternative explanations for their upregulation.

αII spectrin is also 1.6-fold upregulated in the high temperature ovaries (Table 1). This protein is mainly cytoskeletal, forming heterotetramers with β spectrin (Machnicka et al., 2012). In our data β spectrin is present at about the same level as the αII spectrin at 18°C and not differentially expressed between the two conditions (Table S1). The upregulation of αII spectrin may help stabilize the cytoskeleton and plasma membranes at the higher temperature (Machnicka et al., 2012).

### Proteolysis

A number of proteins showing differences in abundance between conditions in our data set can be associated with proteolysis. The most upregulated of these is a protease inhibitor, similar to human serpin B9 (Fig. 7E). In humans this protein inhibits specific enzymes including caspases (Izuhara et al., 2008). This suggests that it may have an anti-apoptotic function. However, the sequences and targets of the different serpins are very similar, so sequence similarity is not a definitive clue to the precise function.

Cathepsin D (Fig. 7F) is the one protease detected as being upregulated in our dataset. It is an intracellular aspartate protease generally localized in lysosomes (Tsukuba et al., 2000; Zaidi et al., 2008). Apart from a major function in protein degradation, it has also been associated with apoptosis (Benes et al., 2008).

While the upregulation of cathepsin D suggests an increase in protein degradation at higher temperature, this is contradicted by the downregulation of two subunits of the proteasome; 26S proteasome non-ATPase regulatory subunit 7 (PSMD7, Fig. 7G) and proteasome subunit beta type-1-B-like (PSMB1, Fig. 7H). The first of these, PSMD7, in absolute quantity is much more reduced than the increase in cathepsin D. Both of these proteins are parts of the 26S proteasome, although PSMB1 is also included in the 20S proteasome (Bedford et al., 2010; Hilt and Wolf, 1996). The decrease in abundance of both these proteasome subunits, along with the increase in the protease inhibitor serpin B9, suggests that intracellular protein degradation overall is reduced at the higher temperature condition.

### Energy metabolism proteins

Examination of the functions of individual metabolic enzymes suggests that those associated with oxidative metabolism and fermentation are relatively downregulated. These include two subunits of ATP synthase, creatine kinase B, and L-lactate dehydrogenase (Table 2). Only one oxidative metabolism enzyme is upregulated - NADH dehydrogenase 1α. Taken together these data suggest that energy metabolism is either downregulated at the higher temperature, or that lower levels of these enzymes are required due to temperature related changes in enzyme kinetics.

## DISCUSSION

### Shotgun proteomic approach

Proteins are the direct mediators of physiological changes in an organism. Therefore, proteomic measurements should be the most direct means of assessing physiological responses to environmental factors at a molecular level. Recent advances in proteomic technology enable the ‘shotgun’ approach that we have used here to identify and quantify more than 1500 proteins in *C. intestinalis* ovarian tissue samples. A debate is ongoing as to whether transcriptional or post-transcriptional regulation is more important in determining physiological protein abundances (Plotkin, 2010; Vogel and Marcotte, 2012). Recent studies have argued that, with the proper analysis, transcriptomic measurements are consistent with eventual protein expression levels (reviewed in Li and Biggin, 2015). On the other hand, physiological systems under stress may be more governed by post-transcriptional mechanisms (Liu and Aebersold, 2016). Certainly RNA-seq technology is capable of sequencing the transcriptome to a much greater depth than shotgun proteomics is able to sequence the proteome. In addition, the huge number of sequencing reads in a single next-generation sequencing run allows for multiplexing, so that in one study more experimental conditions and/or replicates are feasible. However, the transcriptome is still many regulatory steps away from the proteome, so we have chosen to take the more direct approach of shotgun LC-MS/MS for this study.

The shotgun MS approach has certain advantages over the widely used 2D gel methods. One advantage is that very small amounts of protein are required. In our case we had ample protein from a single *C. intestinalis* ovary, a few mm in diameter, for an LC-MS/MS run. By comparison, a 2D gel experiment requires more protein, eliminating the possibility of tissue specificity (Serafini et al., 2011). Another advantage to the label-free quantitative shotgun approach is that one obtains a list of all detectable proteins in the sample and their abundances, not just those with apparent differences in expression level. These more extensive data allow for a better picture of the global proteome.

Because of the limitation in the number of replicate MS runs that we could practically accomplish, our experimental design had just one acclimation aquarium at each of the two temperatures, and we sampled five individuals from each aquarium. This design risks confounding aquarium effects, such as differences in the microbes or pathogens that might develop in an individual aquarium with effects due strictly to the temperature differences. Ideally one would have multiple completely independent aquaria at each temperature condition, and take replicate protein samples in turn from those independent aquaria (Cornwall and Hurd, 2016; Dowd, 2012; Hurlbert, 1984). We sought to minimize these risks by having a common head tank feeding running seawater and food to the two aquaria.

### Comparison with acute heat-shock findings

The most similar study to the present one is the survey of the heat shock induced proteome of the two *Ciona* congeners, *C. intestinalis* and *C. savignyi* (Serafini et al., 2011). This study, like ours, measures temperature effects on the proteome of the common ascidian *Ciona.* It differed from the present study in that it measured acute temperature effects rather than chronic effects. In addition, Serafini et al. used the 2D gel approach coupled with MALDI-TOF/TOF spectrometry. This resulted in much reduced sequencing depth compared with our study. Finally, their study used whole animal protein samples, while ours used only ovaries. However, several of their findings may be compared and contrasted with ours; although in this study we focus on *C. intestinalis* results only.

First, both studies found expression of the heat shock proteins Hsc71 and Hsp90. In the case of our chronic high temperature condition, we found expression of Hsc71 significantly upregulated in the high temperature condition, while Hsp90 is expressed at a low level in both conditions. Serafini et al. did not find significant differences in the expression of these large HSPs for *C. intestinalis*, and suggested that the HSPs were already expressed in the normal temperature condition preemptively guarding against stress. Our measurements are consistent with this view, in that Hsc71 is abundant at the control temperature, as expected since it is a constitutive Hsp70 (Mayer, 2013), becoming coordinatively upregulated at the elevated temperature. Our finding of upregulation of Hsc71 at the higher temperature may reflect a difference between the acute heat shock of Serafini et al. and the chronic temperature elevation of our study. Since neither study found upregulation of Hsp90 or other Hsp70s, it may require a larger temperature difference to see expression changes for these inducible Hsps.

Serafini et al. also found significant upregulation of extracellular matrix (ECM) proteins due to heat shock which we did not detect. They also found evidence for remodeling of the cytoskeleton, as in the present study, although the particular changes in protein expression differ.

### Possible role of the endoplasmic reticulum

Many of the proteins found differentially regulated are associated, or potentially associated, with the endoplasmic reticulum (ER), suggesting that the ER is central to the response to temperature changes. Three cellular functions are particularly implicated: protein folding (or the unfolded protein response), calcium homeostasis, and vesicle transport. The two upregulated protein disulfide isomerases (PDIs), and the downregulated peptidyl-prolyl cis-trans isomerase are likely active in the ER (Braakman and Hebert, 2013; Ellgaard and Ruddock, 2005). Glutathione peroxidase is an important catalyst for oxidation of PDIs, so its upregulation may be coordinated with that of the PDIs (Bulleid, 2012). In addition, the two upregulated HSPs may function in part in the ER. Calcium homeostasis is largely regulated by the ER, and CAB39, EFHD2, and EFHD8 likely reside there. Since the ER is the main site for lipid biosynthesis in the cell, both the highly upregulated transport proteins sec1/SNARE binding protein and ApoB may be important for intracellular and intercellular lipid transport from the ER (English and Voeltz, 2013).

### A model for the high temperature response in the *C. intestinalis* ovary

**Fig. 8.**
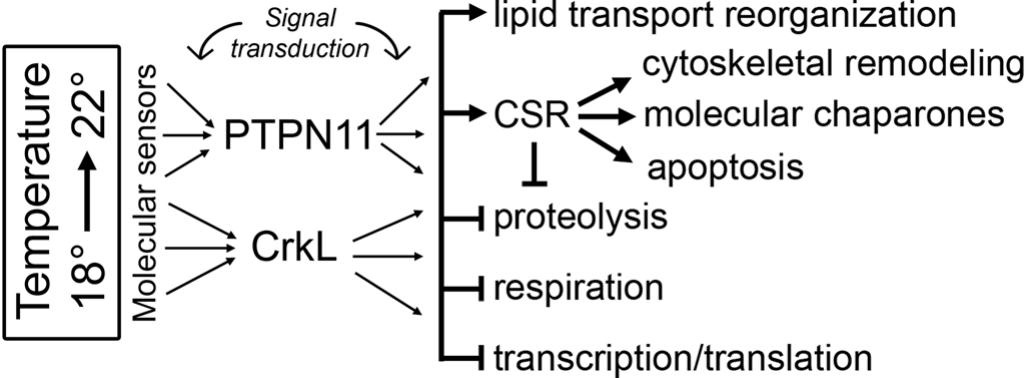
**A model for the elevated temperature proteomic response in *C. intestinalis.*** Refer to the text for explanation.

As outlined above, many of the differentially regulated proteins identified here are associated with a cellular stress response, suggesting that the ovaries are experiencing temperature stress at the higher temperature. However, it is also possible that the differences in protein expression have more to do with accelerated growth and development due to the temperature increase. Determining which of these processes account for the different changes in protein expression that we have measured will require additional investigation. This work may need to involve manipulation of individual protein levels, and/or measurement of indicators of stress, to assess the extent to which the temperature acclimation is actually stressing cells versus the cells responding to temperature by changing homeostatic conditions. In either case, the proteomic data suggest several trends in protein expression related to elevated water temperature. Fig. 8 depicts a model for how these trends might relate to each other. While speculative in terms of mechanism, the model provides a testable set of working hypotheses for how the ovary responds to elevated temperature.

Acclimation to a change in water temperature from 18 to 22°C induces an extreme upregulation of the signaling molecules PTPN11 and CrkL (114- and 27-fold, respectively). As mentioned above, both of these proteins participate in assembly of signal transduction complexes, and they are structurally related in that they share SH2 protein binding domains (Tajan et al., 2015; Neel et al., 2003; Feller, 2001). In at least one pathway they are common interaction partners with Gab1, a substrate of the c-Met receptor tyrosine kinase (Schaeper et al., 2000). We propose that this indicates PTPN11 and CrkL participate in a key signal transduction pathway, or pathways, leading to five main effects.

First, they activate reorganization of lipid transport. As mentioned above, *C. intestinalis* has three members of the large lipid transport family: ApoB, which is highly upregulated, vitellogenin, which is downregulated, and MTTP, which is not differentially regulated. ApoB binds lipid-containing vesicles directly, presumably in this case for yolk transport. Another lipid transport protein, sec1, is also among the most highly upregulated proteins. This molecule has been most associated with vesicle transport and membrane fusion through syntaxin-1.

The second major trend in the data is modification of the CSR (Kültz, 2005; Tomanek, 2014). The CSR is manifested in part through upregulation of molecular chaperones - including Hsc71, Hsp22, and two protein disulfide-isomerases (PDIA-TMX3 and PDIA1/3/4). In addition, the antioxidant glutathione peroxidase is upregulated. Other stress response proteins are downregulated at the higher temperature (Fig. 4D-H) suggesting that the ovary is reallocating energy to a different aspect of the CSR at 22°C versus 18°C. Temperature change is also associated with remodeling of the cytoskeleton (Fields et al., 2012; Madeira et al., 2016; Serafini et al., 2011). This effect is seen in our data in upregulation of β-tubulin and tropomyosin, and downregulation of α-tubulin and cofilin (Fig. 7A-D). Another aspect of the CSR is activation of apoptosis. There is weak evidence in the data that apoptosis may be upregulated at higher temperature. Two of the upregulated proteins, IGF2BP1 and Hsc71, are associated with the GO term ‘apoptosis’ in a PANTHER search. None of the downregulated proteins had that designation.

A third trend in our data is the apparent downregulation of proteolysis at the higher temperature. This is most clearly indicated by the reduced abundance of two subunits of the 26S proteasome, and the upregulation of the protease inhibitor serpin B9 (Fig. 7E). Inhibition of proteolysis is somewhat unexpected at the higher temperature condition, which might be expected to reduce the stability of proteins. The apparent downregulation of proteolysis may be a case of hormesis, where stresses up to a certain point actually increase physiological performance by activating stress response mechanisms (Costantini et al., 2010).

Fourth, proteins involved in cellular respiration, such as ATP synthase and L-lactate dehydrogenase are among those downregulated at the higher temperature. This is consistent with some other cases of temperature stress, which may be the organism shifting energy from respiration to the CSR to protect cells from damage (Fields et al., 2012, 2015; Hofmann and Todgham, 2010), and in particular from reproduction to the CSR (Petes et al., 2008). It is also possible that enzyme kinetics are accelerated at the higher temperature allowing for lower levels of respiratory enzymes to achieve homeostasis.

Finally, certain transcription, post-transcriptional processing, and translation-related proteins are downregulated at the higher temperature (Fig. 6). In particular, reduced abundance of RNA polymerase I points to reduced production of ribosomes and downregulation of protein synthesis. In addition, the post-transcriptional processing proteins RBPMS and MBNL are downregulated, which may indicate the slowing of transcription generally. These data are contradicted, however, by upregulation of other transcription-related proteins (Fig. 6A-C). As with respiration it could be that different levels of these proteins are required due to changes in enzyme kinetics at the higher temperature.

This hypothesis focusing on PTPN11 and CrkL is certainly overly simplified, since the temperature-dependent response undoubtedly involves expression and post-translational modification of multiple regulatory proteins apart from these two. However, it provides an initial testable prediction that knock-down or knock-out of PTPN11 would reduce the differential protein expression effects seen in the present study. We also predict that knockout of PTPN11, produced perhaps by CRISPR/Cas9 genome editing, would lead to a severe effect on reproduction at elevated temperatures. A possible problem would be that knockout of PTPN11 would have a severe effect at the control temperature of 18°C as well, confounding the analysis, even though it is expressed at a very low level in ovaries. One possibility for avoiding this would be to knock down rather than completely ablate expression of PTPN11 using CRISPR-interference mediated transcriptional repression or short-interfering siRNA (Gilbert et al., 2013; Chen et al., 2014).

Such an experiment is feasible in *C. intestinalis* given the abundant bioinformatic resources and technical background available, including CRISPR/Cas9 protocols (Sasaki et al., 2014; Stolfi et al., 2014). Reproduction can be assayed by monitoring fertilization and development *in vitro*. Gene expression can be globally assayed by either RNA-seq or MS/MS proteomics. Coupling these technologies will provide powerful tools for discovering the molecular physiological effects of temperature changes in marine organisms such as *C. intestinalis.*

## MATERIALS AND METHODS

### Animal culture

*C. intestinalis* gametes were obtained from two adults collected at Point Judith Marina in Point Judith Pond in southern Rhode Island (41.387°N, 71.517°W). The eggs from both adults were mixed and fertilized *in vitro* with mixed sperm from the same two adults. The offspring were settled on acrylic plates at 15°C and over the course of a week brought up to 18°C in a programmable environmental chamber. The plates were transferred to two 150-liter tanks with flowing seawater. One tank was maintained at 18°C, while the other was gradually raised over the course of two weeks to 22°C and maintained at that temperature. Unfiltered ambient temperature raw seawater was added by means of a head tank, which fed both rearing tanks at an equal rate of approximately 1 liter/min. Constant temperature was maintained in the rearing tanks by way of individual heaters and chillers as required.

### Tissue preparation

After a total rearing time of 120 days, adult animals were spawned by light cycling and cross-fertilized with sperm from another animal. Immediately afterwards, ovaries were dissected from adults at each temperature, frozen in liquid nitrogen and stored at −70°C. Ovaries from five adults from each condition with gametes that developed normally, were chosen for proteomic analysis. The ovary tissue was washed with PBS and homogenized with 9 M Urea buffer (9 M urea; 20 mM HEPES, pH 8) for 1 min in a glass douncer. The samples were sonicated using a Fisher Scientific Sonic Dismembranator model 500 at 10% amplitude for 5 s and placed on ice for 30 s; this step was repeated six times. The samples were centrifuged at 20,000×***g*** at 15°C for 15 min, and the supernatant lysates were frozen and delivered to the Proteomics Core of the COBRE Center for Cancer Research and Development at Rhode Island Hospital, in Providence, RI, USA where trypsin digestion, quantification, and LC-MS/MS were performed.

### LC-MS/MS analysis

LC/MS was performed as described previously (Nguyen et al., 2009). Tryptic peptides were analyzed by a fully automated proteomic technology platform (Yu and Salomon, 2009, 2010). The nanoLC-MS/MS experiments were performed with an Agilent 1200 Series Quaternary HPLC system (Agilent Technologies, Santa Clara, CA, USA) connected to a ‘Q Exactive Plus’ mass spectrometer (Thermo Fisher Scientific, Waltham, MA, USA).

The lyophilized tryptic peptides were reconstituted in buffer A (0.1 M acetic acid) at a concentration of 1 µg/µl and 5 µl was injected for each analysis. The electrospray ion source was operated at 2.0 kv in a split flow configuration, as described previously (Elias and Gygi, 2007). The Q Exactive was operated in the data-dependent mode using a top-9 data dependent method. Survey full scan MS spectra (*m/z* 400-1800) were acquired at a resolution of 70,000 with an AGC target value of 3×10^6^ ions or a maximum ion injection time of 200 ms. Peptide fragmentation was performed via higher-energy collision dissociation (HCD) with the energy set at 28 NCE. The MS/MS spectra were acquired at a resolution of 17,500, with a targeted value of 2×10^4^ ions or a maximum integration time of 250 ms. The under fill ratio, which specifies the minimum percentage of the target value likely to be reached at maximum fill time, was defined as 1.0%. The ion selection abundance threshold was set at 8.0×10^2^ with charge state exclusion of unassigned and *z*=1, or 6-8 ions and dynamic exclusion time of 20 s.

### Data analysis

Peptide spectrum matching of MS/MS spectra from whole cell lysate tryptic digest samples was performed against a *Ciona intestinalis*-specific database (UniProt; downloaded 07/27/2015) using MASCOT v. 2.4 (Matrix Science, Ltd, London, UK). A concatenated database containing 144,156 ‘target’ and ‘decoy’ sequences was employed to estimate the FDR. Msconvert from ProteoWizard (v. 3.0.5047), using default parameters and with the MS2Deisotope filter on, was employed to create peak lists for Mascot. Mascot database searches were performed with the following parameters: trypsin enzyme cleavage specificity, two possible missed cleavages, 7 ppm mass tolerance for precursor ions, 20 mmu mass tolerance for fragment ions. Search parameters permitted variable modification of methionine oxidation (+15.9949 Da), and static modification of carbamidomethylation (+57.0215 Da) on cysteine. The resulting peptide spectrum matches (PSMs) were reduced to sets of unique PSMs by eliminating lower scoring duplicates. Mascot results were filtered by Mowse Score (>20). Peptide assignments from the database search were filtered down to 1% FDR by a logistic spectral score, as previously described (Ficarro et al., 2005; Yu et al., 2009). This method resulted in identification of 1616 proteins found in both the 18°C and 22°C samples.

### Quantitation of relative peptide abundance

Relative quantification of peptide abundance was performed via calculation of selected ion chromatograms (SIC) peak areas. Retention time alignment of individual replicate analyses was performed as previously described (Demirkan et al., 2011). Peak areas were calculated by inspection of SICs using in-house software programmed in R 3.0 based on the Scripps Center for Metabolomics' XCMS package (version 1.40.0). This approach performed multiple passes through XCMS's central wavelet transformation algorithm (implemented in the centWave function) over increasingly narrower ranges of peak widths, and used the following parameters: mass window of 10 ppm, minimum peak widths ranging from 2 to 20 s, maximum peak width of 80 s, signal to noise threshold of 10, and detection of peak limits via descent on the non-transformed data enabled. For cases when centWave did not identify an MS peak, we used the getPeaks function available in XCMS to integrate in a pre-defined region surrounding the maximum intensity signal of the SIC. SIC peak areas were determined for every peptide that was identified by MS/MS. In the case of a missing MS/MS for a particular peptide, in a particular replicate, the SIC peak area was calculated according to the peptide's isolated mass and the retention time calculated from retention time alignment. A minimum SIC peak area equivalent to the typical spectral noise level of 1000 was required of all data reported for label-free quantitation. Individual SIC peak areas were normalized to the peak area of the exogenously spiked synthetic peptide DRVYHPF added prior to reversed-phase elution into the mass spectrometer.

### Statistical analysis

Proteins missing more than one intensity measurement per temperature condition were eliminated from the analysis, leaving 1532 identified proteins. All intensity measurements were log_10_ transformed to reduce the variance between the sample groups. Because the comparisons between temperature conditions were a mixture of normally and non-normally distributed data, we calculated *P*-values using both Student's *t*-test (using Microsoft Excel) and the non-parametric Mann–Whitney *U*-test (using SPSS; IBM Corporation) for the MS peak intensity data. To account for multiple hypothesis testing, the Benjamini-Hochberg method was also used to calculate Q-values. Because of the small number of samples given the many comparisons, the multiple hypothesis criterion was judged to be overly stringent (Dowd, 2012). However, a fold-change cutoff for the means of the two conditions was set based on the Qvalue for Hsc71 (Q=0.036), which had the lowest fold-change of the identified proteins with Q<0.05 according to the Q-value false discovery test. By this criterion the upregulation fold change cutoff was set at a value for 10log_10_>2.0, i.e. >1.58. Conversely, the fold-change cutoff for downregulation of the 22°C condition versus 18°C was set at 10log_10_<−2, i.e. <0.63. These thresholds can be considered inflection points for differentially regulated proteins (refer to Fig. S2). In summary, to be considered differentially regulated a protein had to have a *P* value of <0.05 by both the *t*-test and *U*-test, and be over or under the fold-change thresholds.

SPSS was used for PCA on MS peak intensity measurements for the 62 differentially expressed proteins using promax rotation with Kaiser normalization (Fig. 1). The heat map (Fig. 2) was generated using Morpheus (https://software.broadinstitute.org/morpheus/).

### Assignment of orthology and functional groups to identified proteins

The web application PANTHER (Mi et al., 2013) was used to produce an initial assessment of GO for the differentially regulated proteins. PANTHER overexpression analysis was also performed using the 1532 protein list from the LC-MS/MS data as the reference list (Table S1). This estimate is necessarily incomplete since about 13% of the proteins in each list are unmapped to orthologs or GO terms by the PANTHER application.

To further explore the putative homology and function of the proteins, the UniProt sequences were BLASTed in the ANISEED database of tunicate sequences (http://www.aniseed.cnrs.fr/aniseed/) (Tassy et al., 2010) to confirm the gene model in UniProt, and access GO information. The protein sequences were also submitted to NCBI DeltaBLAST and the best human match identified to aid in annotation of protein function. In addition, the sequences were searched in the INTERPRO database (Mitchell et al., 2015) to supplement the search for conserved protein domains identified in the DeltaBLAST. Our manual categorization of protein functions based on these web searches and cited literature are summarized in Tables 1 and 2.
